# Transcriptome Analysis of the *Trachinotus ovatus*: Identification of Reproduction, Growth and Immune-Related Genes and Microsatellite Markers

**DOI:** 10.1371/journal.pone.0109419

**Published:** 2014-10-10

**Authors:** Xie Zhenzhen, Xiao Ling, Wang Dengdong, Fang Chao, Liu Qiongyu, Li Zihao, Liu Xiaochun, Zhang Yong, Li Shuisheng, Lin Haoran

**Affiliations:** 1 State Key Laboratory of Biocontrol, Institute of Aquatic Economic Animals, and the Guangdong Province Key Laboratory for Aquatic Economic Animals, School of Life Sciences, Sun Yat-Sen University, Guangzhou, China; 2 College of Ocean, Hainan University, Haikou, Hainan, China; Beijing Institute of Genomics, China

## Abstract

**Background:**

The *Trachinotus ovatus* (Teleostei, Carangidae) is an economically important marine fish species in the world. However, the lack of genomic information regarding this species limits our understanding of the genetics and biological mechanisms in *Trachinotus ovatus*. In this study, high throughput transcriptome sequencing was used to obtain comprehensive genomic information in *Trachinotus ovatus*.

**Principal Findings:**

Transcriptome sequencing was performed by using Illumina paired-end sequencing technology. The 98,534,862 high quality reads were yielded, and were *de novo* assembled into 156,094 unigenes with an average sequence length of 1179 bp. Transcriptome annotation revealed that 75,586 and 67,923 unigenes were functionally annotated in the NCBI non-redundant database and Swiss-Prot protein database, respectively. Functional analysis demonstrated that 67,923 unigenes were grouped into 25 Cluster of Orthologous Groups (COG) functional categories, 37,976 unigenes were clustered into 61 Gene Ontology (GO) terms, and 38,172 unigenes were assigned to 275 different Kyoto Encyclopedia of Genes and Genomes (KEGG) pathways. Based on the transcriptome dataset, a large number of unigenes associated with reproduction, growth and immunity were identified. Furthermore, a total number of 38,794 simple sequence repeats (SSRs) were discovered and 16 polymorphic loci were characterized in *Trachinotus ovatus*.

**Conclusion/Significance:**

The present study is the first transcriptome analysis of a fish species belonging to the genus *Trachinotus* and provides a valuable genomic resource for novel gene discovery, gene expression and regulation studies, and the identification of genetic markers in *Trachinotus ovatus* and the other fish of the genus *Trachinotus*.

## Introduction

There are 20 species of carangid fish that belong to the genus *Trachinotus*. They are distributing all over the world and possess very desirable biological characteristics such as fast adaptation to controlled conditions, rapid growth, and so on. Therefore, fish species belonging to the genus *Trachinotus* have long been considered as promising candidates for mariculture [1]. According to the Food and Agriculture Organization of the United Nations (FAO) fishery statistics, the global aquaculture production of the carangid fish had exceeded 113,000 tons in 2012, with a value of approximately 458 million US dollars [2].

*Trachinotus ovatus* (Linnaeus 1758), the most popular cultured species of the genus *Trachinotus*, mainly inhabits in tropical and subtropical waters of the eastern Atlantic Ocean, and is also found in the Mediterranean and along the African coast, including offshore islands [3]. Due to its fast growth rate, pleasing flavor and increased market demand, *Trachinotus ovatus* is recognized as one of the most economically important marine fish species. With the wide use of large-scale marine cage culture in the Asia-Pacific region, including China and Southeast Asian countries, production of this species has increased in momentum in recent years.

However, with the expansion of large-scale culture and the neglect of fishery management and conservation, the genetic diversity of *Trachinotus ovatus* appears to be declining. Several biological changes including early age of sexual maturation, low growth rate, and increasing disease susceptibility have been found in cultured *Trachinotus ovatus* [4]. Since 2001, frequent disease occurrence has been reported in cultured *Trachinotus ovatus* each year in the southern coast of China, resulting in great economic losses [5], [6].

In spite of its significant economic importance, there is very little existing basic research on *Trachinotus ovatus*. Although some DNA markers (amplified fragment length polymorphism and microsatellite markers) have been developed [7], [8], the genetic diversity and population structure of *Trachinotus ovatus* cannot be effectively assessed with the existing genetic information. With the exception of a few studies on growth performance and feeding in confined environments [9]–[11], there is currently only limited information on other important traits such as reproduction, growth and immunity in *Trachinotus ovatus*. Moreover, owing to the lack of genomic data, molecular-genetic association studies remain unexplored in this species. Therefore, all areas of research, particularly the basic research on the genetics and on the gene networks involved in the regulation of reproduction, growth and immune response should be strengthened to support the sustained development of the *Trachinotus ovatus* industry.

Despite the rapid development of high-throughput sequencing technologies, genome sequencing is still expensive and takes long time. Transcriptome sequencing is a good choice for quickly and cost-effectively obtaining large-scale genetic information and functional genes in non-model species that do not have reference genomic information. Transcriptome analysis has now been widely applied in the fields of ecology, genetics, and molecular biology, playing valuable roles in gene discovery, gene expression and regulation, developing molecular markers and so on [12]–[16].

In the present study, using the Illumina paired-end sequencing technology, transcriptome analysis on various tissues of *Trachinotus ovatus* was performed. The genes involved in biological processes associated with reproduction, growth, and immunity were analyzed, and a set of microsatellite markers was developed. Our study provides abundant genomic information for future research into the genetics and molecular biology of *Trachinotus ovatus*.

## Materials and Methods

### Sample Preparation and RNA Extraction

Ten two-year-old *Trachinotus ovatus* with body weight of approximately 1000 ± 100 g were obtained from the Daya Bay Aquaculture Center, Guangdong, China. Fish were anesthetized and sacrificed by decapitation. Tissue samples were collected immediately and snap frozen in liquid nitrogen. All animal experiments were conducted in accordance with the guidelines and approval of the respective Animal Research and Ethics Committees of Sun Yat-Sen University.

Total RNA was isolated from different tissues of female and male *Trachinotus ovatus* (brain, pituitary, liver, gill, heart kidney, kidney, spleen, testes, and ovaries) using Trizol Reagent (Invitrogen, USA) according to the manufacturer's instructions. The concentration of total RNA was estimated by measuring the absorbance at 260 nm using a 2100 Bioanalyzer (Agilent Technologies, USA), and the RNA integrity was checked by ethidium bromide staining of 28S and 18S ribosomal bands on a 1% agarose gel. Equal volumes of RNA from each tissue were pooled and used for cDNA synthesis.

### cDNA Library Construction and Sequencing

The cDNA library construction and sequencing was carried out as described by Li [17]. Briefly, Poly (A) mRNA was isolated from 20 ug of total RNA using oligo-dT beads (Qiagen, Germany), and was broken into short fragments (200 nt) in fragmentation buffer. The short fragments were used to synthesize first-strand cDNA by using random hexamer-primed reverse transcription, and then the second-strand cDNA was synthesized using RNase H and DNA polymerase I. The double-stranded cDNAs were purified using the QIAquick PCR extraction kit (Qiagen, Germany). After washing with EB buffer, the purified products were used for end reparation poly (A) addition and ligated to sequencing adapters. Following agarose gel electrophoresis, the adaptor-ligated fragments (200 bp ± 25 bp) were further extracted on the agarose gel and enriched by PCR to construct the final cDNA library, which was sequenced on the Illumina HiSeq 2000 sequencing platform using single-end paired-end technology in Beijing Genomics Institute-Shenzhen, Shenzhen, China. The original data process to sequences, base-calling and quality value calculation were achieved by the Illumina Genome Analyzer Pipeline (version 1.6), and 100 bp paired-end reads were obtained.

### Illumina Reads Processing and Assembly

A Perl program was written to select clean reads. Low-quality reads that were more than 50% bases with quality lower than 20 in one sequence, ambiguous reads containing more than 5% unknown bases, and reads containing adaptor sequences were removed. Then the clean reads were assembled using Trinity to construct unique consensus sequences [18].

### Functional Annotation and Classification

Using the BLASTx tool which was developed to evaluate the similarities between two sequences [19], the filtered transcripts were search against the National Center for Biotech-nology Information (NCBI) non-redundant protein (Nr) database (http://www.ncbi.nlm.nih.gov/), the Swiss-Prot protein database (http://www.expasy.ch/sprot) [20], the Cluster of Orthologous Groups (COG) (http://www.ncbi.nlm.nih.gov/COG/) [21], and the Kyoto Encyclopedia of Genes and Genomes (KEGG) pathway database [22] with an E-value cut off of 1e-5. Protein sequences from the databases which had the highest similarity scores were used as the functional annotation for the related unigene. Blast2GO was applied to get the annotation results of unigenes in Gene Ontology (GO) database (http://www.geneontology.org/) [23]. The WEGO software (http://wego.genomics.org.cn/cgibin/wego/index.pl) [24], a statistics tool, was then used to classify the annotation results of unigenes in GO database. Meanwhile, the unigenes were also aligned to the KEGG database to annotate the signal pathways.

### Simple Sequence Repeat (SSR) Detection and Primer Design

Potential SSR markers were detected among the 156,094 unigenes using the MIcroSAtellite identification tool (MISA) (http://pgrc.ipk-gatersleben.de/misa/) [25]. We searched for SSRs with motifs ranging from di- to hexa-nucleotides in size. Given difficulties in distinguishing genuine mono-nucleotide repeats from polyadenylation products and some mono-nucleotide repeats that were generated by base mismatches or sequencing errors, mono-nucleotide repeats were discarded in this study. According to the MISA results, primer pairs flanking each SSR locus were designed using the Primer3 program (http://www.broadinstitute.org/genome_software/other/primer3.html) [26].

### Survey of SSR Polymorphism

Fin samples were randomly collected from 30 six-month old *Trachinotus ovatus* individuals, which were the progeny of one broodstock population in the Daya Bay Aquaculture Center, Guangdong, China. All the samples were placed in absolute ethanol and kept frozen at −20°C until DNA extraction. Polymorphism was tested in the 30 *Trachinotus ovatus* individuals. Genomic DNA was extracted from the fin samples using the salting-out procedure [27]. PCR was performed on a Veriti Thermal Cycler in a total volume of 20 uL containing 0.4 uM of each primer, 10× PCR buffer (Genstar, China), and 100 ng DNA. Cycling conditions consisted of initial denaturation at 94°C for 5 min, 35cycles of 45 s at 94°C,40 s at the annealing temperature, 40 s at 72°C, and a final cycle of 5 min at 72°C. Allele sizes were estimated according to the pBR322 DNA/MspI marker (TianGen, China) after PCR products were separated on 8% denaturing polyacrylamide gel. The expected and observed heterozygosities together with an analysis of Hardy-Weinberg equilibrium (HWE) and linkage disequilibrium were calculated using GENEPOP 4.0 [28]. Null allele frequencies were calculated with MICRO-CHECKER2.2.3 [29]. The significant values for all multiple tests were corrected by the sequential Bonferroni's procedure [30]. Polymorphism information content (PIC) was calculated using the PIC-CALC 0.6 software.

## Results and Discussion

### Sequencing, Assembly and Evaluation

Using the Illumina sequencing platform, a total of 99,714,868 raw reads were produced. After strict quality control and data filtration, 98,534,862 cleaned reads were harvested, with an average length of 100 bp (Table 1). All cleaned reads generated in this study have been deposited in the NCBI Sequence Read Archive (SRA) database (accession number: SRA161953).

**Table 1:** Statistics of the assembled transcripts and unigenes.

	Total number	Total Nucleotides base pair (bp)	Average length(bp)	N50
Raw Reads	99,714,868	9,971,486,800	100	–
Clean Reads	98,534,862	9,853,486,200	100	–
Unigenes	156,094	184,107,954	1179	2404

**Figure 1:**
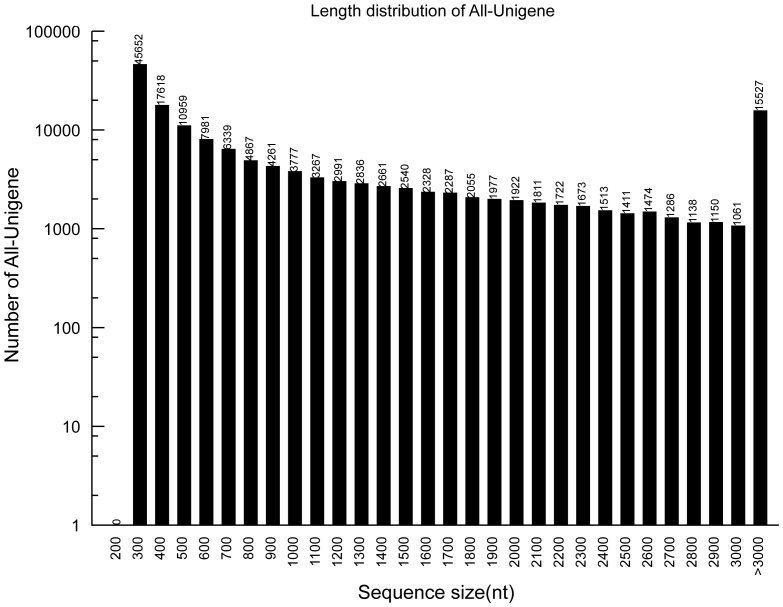
Length distribution of all unigenes of *Trachinotus ovatus*.

Using the Trinity assembly program, 156,094 unigenes were generated by *de novo* assembly, with an average length of 1179 bp and an N50 of 2404 bp (Table 1). The mean length of the unigenes in this study was longer than those assembled in other teleost fish such as *Misgurnus anguillicaudatus* (387 bp) [31], *Oncorhynchus mykiss* (662 bp) [32], *Scophthalmus maximus* (671 bp) [33] using *de novo* transcriptome assembly or 454 sequencing. Furthermore, the number of assembled unigenes and their length distribution were also analyzed. About 53,930 unigenes were >1,000 bp in length (Figure 1).

**Figure 2:**
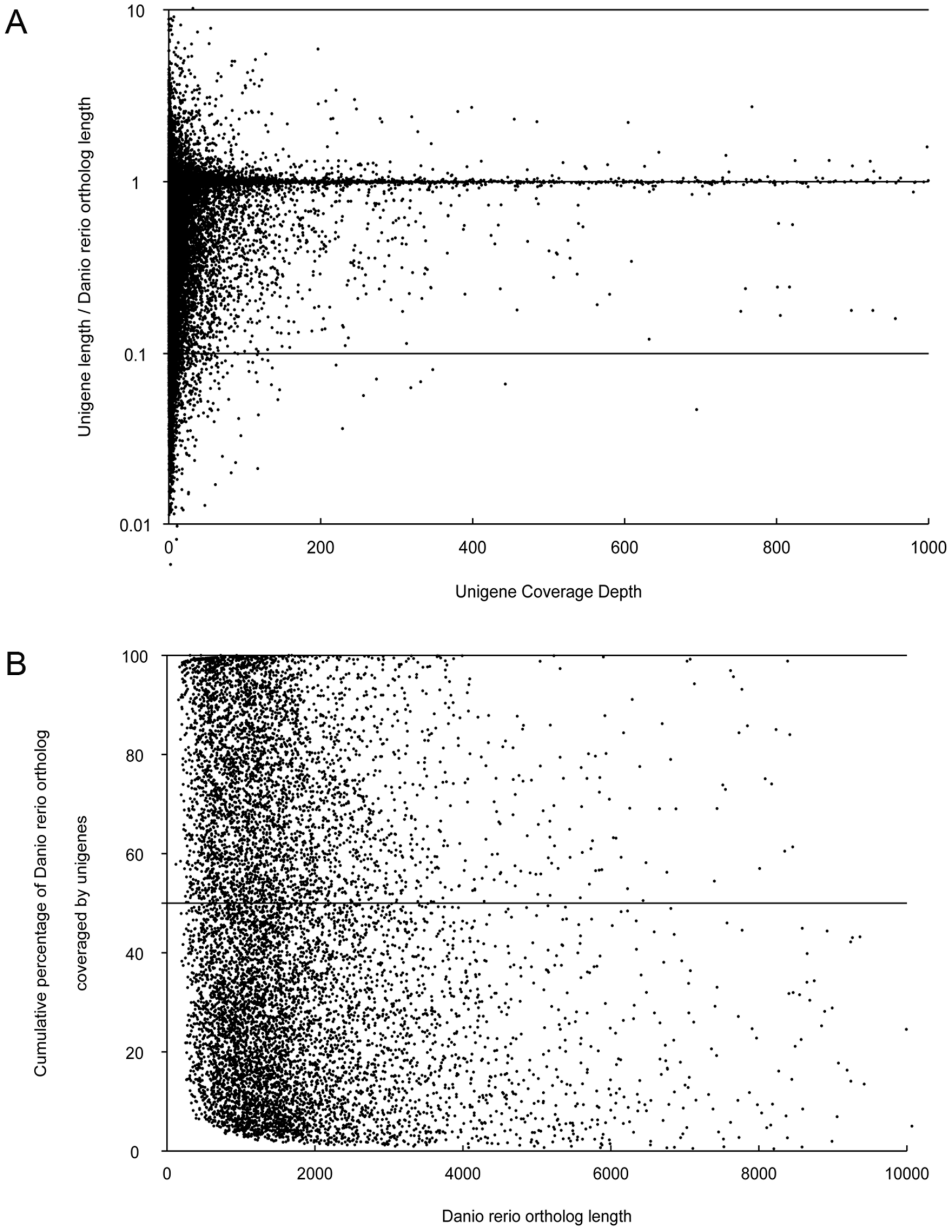
Comparison of *Trachinotus ovatus* unigenes to *Danio rerio* orthologous coding sequences. (A) The ratio of *Trachinotus ovatus* unigene length to *Danio rerio* ortholog length was plotted against *Trachinotus ovatus* unigene coverage depth. (B) The total percentage of *Danio rerio* ortholog coding sequence covered by all *Trachinotus ovatus* unigenes.

In order to assess the extent of coverage of the assembled unigenes and of evaluating the effects of coverage depth on unigene assembly, the ratio of the assembled unigene length to *Danio rerio* ortholog length against coverage depth was calculated. As shown in Figure 2A, the coding regions of most of *Danio rerio* orthologs could be effectively covered by the assembled *Trachinotus ovatus* unigenes. There were 10,219 unigenes with the ratio within 0.8 to 1.2, providing abundant long transcripts for gene function studies in *Trachinotus ovatus*. Additionally, it was observed that coverage depth was not directly proportional to coverage of the coding regions, although increased coverage depth could improve the extent of transcript coverage to a certain degree. The total percentage of *Danio rerio* ortholog coding sequences that were covered by all *Trachinotus ovatus* unigenes was also plotted. As shown in Figure 2B, 1,679 *Danio rerio* orthologs could be covered by *Trachinotus ovatus* unigenes with a percentage more than 80%. In addition, there were about 2,337 *Danio rerio* orthologs with coverage varying from 50% to 80%, and 2,725 orthologs with coverage below 20%. These data indicate that the assembled unigenes in this current study are of high quality of the present assembly.

### Annotation of Unigenes

**Figure 3:**
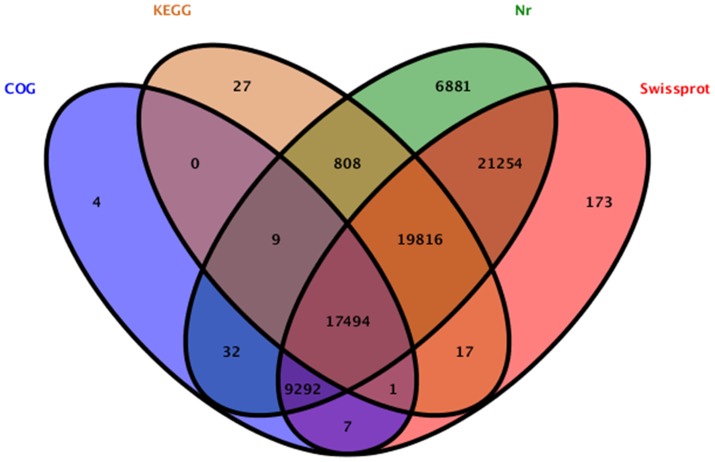
Venn diagram of annotation results against Nr, SwissProt, COG and KEGG databases. The number in each color block indicates the number of unigenes that is annotated by single or multiple databases.

Using the BLASTx algorithm (E-value <10^−5^), all assembled unigenes was searched against the databases of NCBI Nr, Swiss-Prot, COG, and KEGG. The annotation results were demonstrated through the Venn diagram (Figure 3). Of the 156,094 unigenes, 75,586 and 67,923 had homologous sequences in the Nr and Swiss-Prot protein databases, while 27,798 and 38,127 unigenes could be classified by COG and KEGG databases, respectively. In total, 15,145 unigenes could be simultaneously annotated by all four databases.

**Figure 4:**
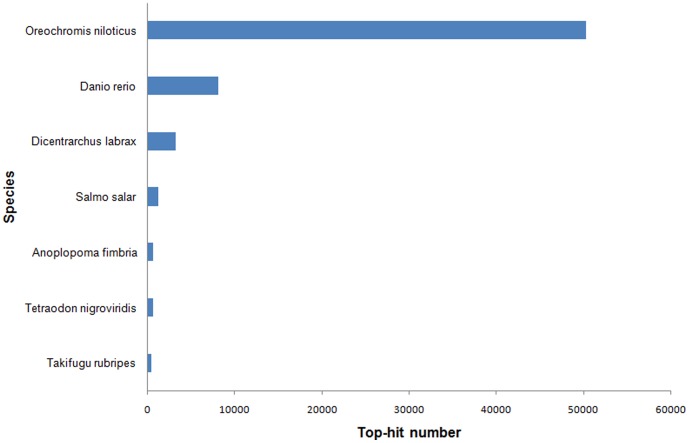
Top-hit species distribution for sequences from *Trachinotus ovatus* submitted BLASTX against the NCBI-Nr database. Only fish species were present in this study.

A BLASTx top-hit species distribution showed that 50,324 unigenes exhibited similarity to the sequences of *Oreochromis niloticus*, 8,173 to the sequences of *Danio rerio*, 3,322 to the sequence of *Dicentrarchus labrax*, and 1,342 to the sequence of *Salmo salar* (Figure 4). These results reveal that the gene content is phylogenetic conserved between *Trachinotus ovatus* and *Oreochromis niloticus*, and indicate that *Trachinotus ovatus* is more closely related to *Oreochromis niloticus*, both of which belong to the order Perciformes.

### COG, GO and KEGG Classification

**Figure 5:**
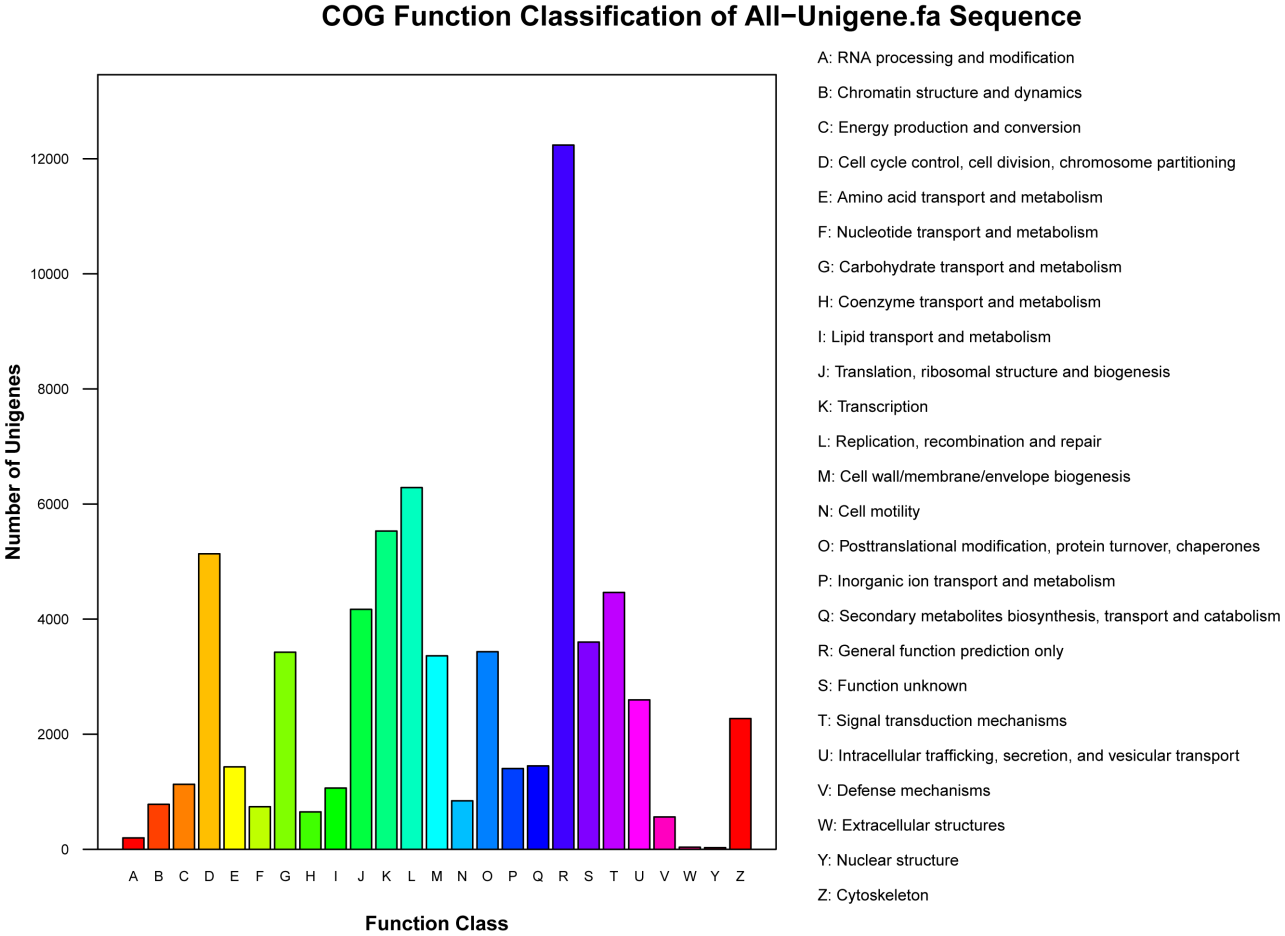
Clusters of Orthologous Groups (COG) functional classification of the *Trachinotus ovatus* transcriptome.

For functional prediction and classifications, all unigenes were aligned to the COG database. Together, 67,923 unigenes were grouped into 25 COG classifications (Figure 5). The largest cluster was the general function prediction only (18.31%), followed by replication, recombination and repair (9.41%), transcription (8.28%), cell cycle control, cell division and chromosome partitioning (7.68%), signal transduction mechanisms (6.68%), translation, ribosomal structure and biogenesis (6.24%), function unknown (5.39%), posttranslational modification, protein turnover, and chaperones (5.14%) and carbohydrate transport and metabolism (5.13%) (Table S1).

**Figure 6:**
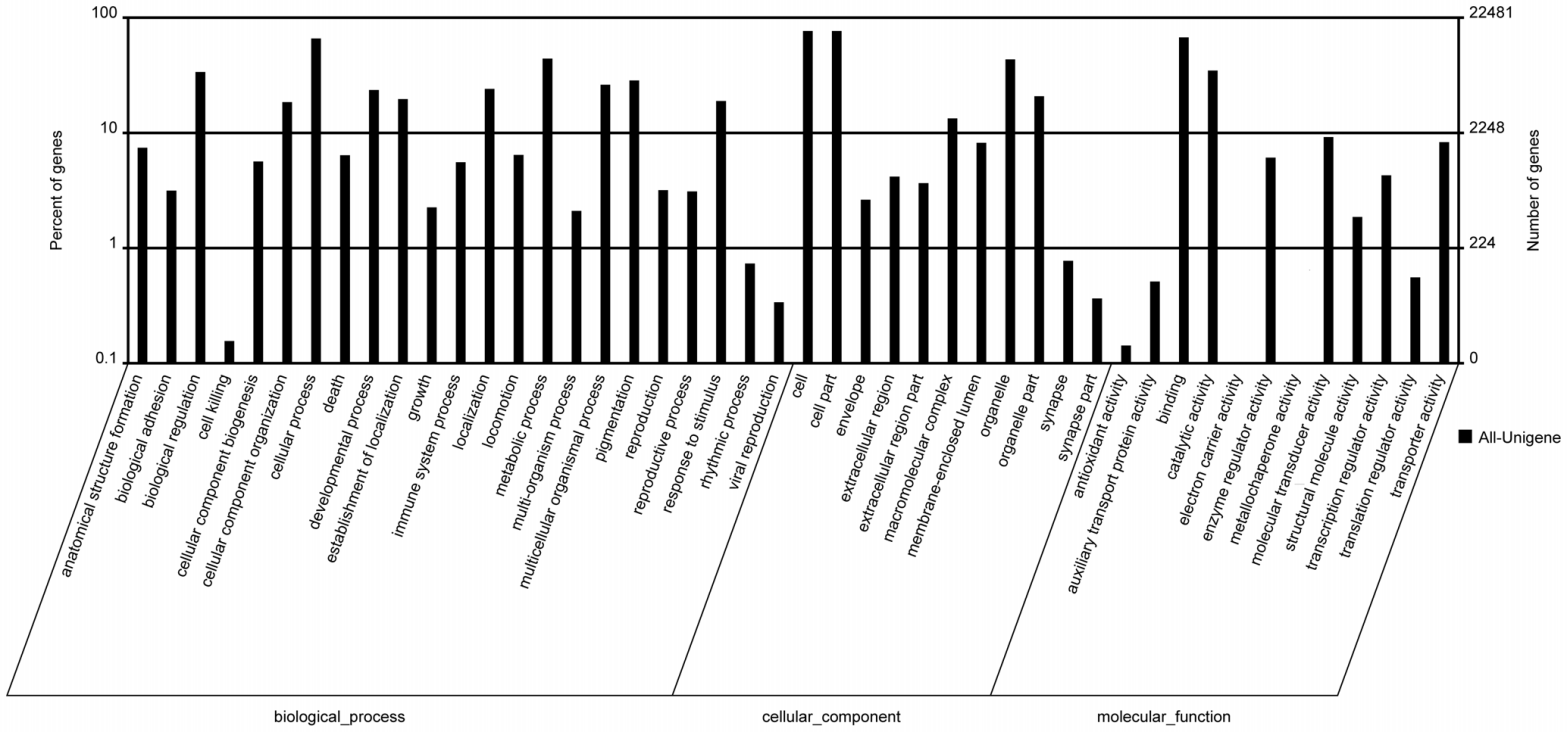
Gene Ontology (GO) analysis and functional classification of the *Trachinotus ovatus* transcriptome.

Gene Ontology is a standardized system for gene functional classification, containing three domains which are classified by biological process, cellular components and molecular functions of gene products [34]. Gene ontology analysis of our dataset showed that a total of 22,481 unigenes were annotated. Of these, 17,838 were grouped into cellular component, 18,064 were grouped into molecular function, and 18,823 were grouped into biological process (Figure 6). In the category of biological process, a large proportion of unigenes were related to cellular progress and metabolic progress. Within the category of cellular component, unigenes related to cell and cell part represented the largest clusters. Under the category of molecular function, a high percentage of unigenes was involved in binding and catalytic activity.

For further identification of the biological pathways in *Trachinotus ovatus*, we mapped the assembled sequences to the reference of typical pathways in the KEGG database. The 38,172 unigenes were matched to 275 different KEGG pathways (Table S2). Among these sequences, 6,639 were classified into metabolism groups, mostly involving in purine metabolism (776), glycerophospholipid metabolism (409), pyrimidine metabolism (358), and glycerolipid metabolism (341). The second largest cluster is the genetic information processing (5,364), involving degradation (1,146), repair (247), transcription and replication (172) and so on. There were 14,981, 9,849, and 1,339 sequences that were classified into cellular processing, human disease and environment information, respectively.

### Identification of Sequences Related to Reproduction

Fish reproduction is the precondition for large-scale commercial culture and breeding. The artificial reproductive manipulation in *Trachinotus ovatus* has been established. However, the regulatory mechanisms behind reproductive development in this species are poorly understood and there is a lack of effective techniques for reproductive manipulation, leading to the abuse of hormone in current culture practices. Reproductive activities in fish are mainly controlled by gonadotropin-releasing hormone (GnRH)-gonadotropic hormone (GtH) axis [35]. GnRH agonists and exogenous GtH preparations have been widely used to induce ovulation and spermiation in cultured fish [36]. Therefore, understanding the regulation of GnRH and GtH is highly important for understanding reproductive activities in *Trachinotus ovatus*. In the transcriptome data, the transcripts for three subtypes of GnRH and different GtH subunits, including the complete coding DNA sequence of follicle-stimulating hormone subunit beta, luteinizing hormone subunit beta were indentified (Text S1). Moreover, the transcripts for kisspeptin, tachykinin3, aromatase, estrogen receptors, and androgen receptors were also found (Text S1). Kisspeptin and tachykinin3 are considered to be the key regulators of GnRH secretion in vertebrates [37]. Aromatase, estrogen receptors, and androgen receptors are critical factors in gonad responding to the actions of GnRH and GtH [38]. These sequences provide valuable information for elucidating the regulatory mechanisms of reproductive axis in *Trachinotus ovatus*. Further studies will likely focus on the role of the GnRH-GtH axis in the regulation of reproductive activities and on establishing highly effective techniques for the induction of gamete maturation, ovulation, and spermiation in *Trachinotus ovatus*.

In addition, *Trachinotus ovatus* is a unique species without any obvious phenotypic characteristics that can be used to distinguish the males and females. Difficulty in identifying the sexes has greatly hampered the artificial breeding program. In order to isolate sex-specific molecular markers, identification of genes related to sex determination and differentiation is necessary. Through the GO analysis, there were about 714 unigenes related to the reproductive regulation (Table S3), including forkhead box L2 that and doublesex and mab-3 related transcription factor 1 (DMRT1), which are key genes in the control of sex determination and differentiation in vertebrates [39]. The Realtime PCR analysis revealed that the expression of DMRT1 was much higher in the testes than in the ovaries (Figure S1), which is consistent with the findings in other fish species [40]–[42]. Studies have found that overexpression of DMRT1 could induce a female-to-male sex-reversal in Nile tilapia [43]. A linkage map in zebrafish has revealed the sex determination locus harboring DMRT1 gene [44]. Therefore, studying the roles of DMRT1 and other sex related genes in sex determination and differentiation will be useful in finding sex-specific markers that can help solve the problem of sex identification in *Trachinotus ovatus*.

### Identification of Sequences Related to Growth and Metabolism

Improving the growth rate of cultivated fish and selecting fish with desired traits including rapid growth are major objectives in aquaculture. Elucidation of the regulatory mechanisms behind growth control will help achieve these goals. Similar to other vertebrates, fish growth is also controlled by growth hormone/insulin-like growth factor I (GH/IGFI) axis [45]. In this study, the genes coding for GH, IGFs and their receptors were identified in the transcriptome of *Trachinotus ovatus* (Text S2). Meanwhile, the signal transducers associated with GH/GHR signaling pathway and IGF/IGFR signaling pathway were also found (Text S2, Table S4). Furthermore, we also discovered genes that regulate GH and IGFs, such as the growth hormone releasing hormone (GHRH), pituitary adenylate cyclase-activating polypeptide (PACAP), somatostatins, insulin-like growth factor-binding proteins (IGFBP) (Text S2). GHRH and PACAP are the main regulators that promote GH release, while somatostatins play an inhibitory role [46]. IGFBP act as the carrier proteins for IGF [47]. With these sequences, a full view of the endocrine regulatory network of growth axis in *Trachinotus ovatus* can be constructed. Nevertheless, further molecular biology and biochemical studies are required to confirm the roles that these gene products play in *Trachinotus ovatus* growth.

Growth is a highly complex process that involves the regulation of appetite, muscle growth, weight gain and, protein and lipid metabolism. We found a few genes related to appetite and muscle development, including neuropeptide Y (NPY), pro-opiomelanocortin (POMC), leptin and myostatin (Text S2). NPY and POMC exert opposite roles in stimulating or inhibiting feeding, respectively [48]. Leptin has been recognized to regulate energy intake and energy expenditure [49]. Myostatins act as negative regulators of muscle growth [50]. Polymorphisms in myostatin genes have been identified in several fish species and are associated with growth traits [51]–[52]. These may be developed to growth-related markers for the molecular marker-assisted breeding.

In addition, signaling pathways involving carbohydrate, protein, and lipid metabolism were found in the KEGG pathway analysis (Table S2). Studies of these metabolic signaling pathways in combination with previous studies on growth performance and digestion [9]–[11] will expand our understanding of growth control in *Trachinotus ovatus* and will help optimize the artificial feeding for its aquaculture.

### Identification of Sequences Related to Immunity

Aquaculture practices have been found to reduce genetic variability in hatchery-reared stocks, which may result in loss of disease resistance [53]–[54]. In recent years, frequent outbreaks of infectious diseases in fish farming have become a great threat to the *Trachinotus ovatus* industry [5]–[6]. However, we have little knowledge on the immune system of this species. In order to establish efficient defenses against pathogenic infections, the first step is to understand the immune system of *Trachinotus ovatus* and to identify genes and pathways involved in its immune response. GO classification revealed that 4,249 and 1,250 unigenes belonged to two subcategories: response to stimulus and immune system process respectively (Table S5, Table S6). KEGG pathways analysis revealed 29 immune-related pathways, including the toll-like receptor (TLR) signaling pathway, antigen processing and presentation, intestinal immune network for Immunoglobulin A (IgA) production, natural killer cell mediated cytotoxicity and so on (Table S7). These signaling pathways provide comprehensive information for the understanding of the *Trachinotus ovatus* immune system.

The toll-like receptor family is an important group of pattern-recognition receptors which are expressed on antigen-presenting cells, participating in innate immune responses and the subsequent promotion of adaptive immune responses [55]. We identified 10 different TLR transcripts in our transcriptome dataset. Moreover, we also found the genes belong to the TLR signaling pathway, such as myeloid differentiation factor 88 (Text S3). The major histocompatibility complex (MHC) are important molecules that help the immune system recognize foreign substances by binding peptide fragments derived from pathogens and presenting them to T cells [56]. Transcripts encoding MHC class I and II were also identified in the transcriptome dataset (Text S3). MHC genes have been considered as candidate markers associated with disease resistance as well, as it were found to be highly polymorphic in teleosts [57]–[59]. Further analysis of TLR and MHC genes will provide insights into *Trachinotus ovatus* immune defense. Additionally, some transcripts coding for cytokines were discovered in this study, including 18 different interleukins (Text S3). Cytokines play important roles in mediating immune responses in vertebrates. Many recombinant cytokines have been approved for clinical use [60], and these small soluble proteins also have the potential to be used as immunostimulants in aquaculture. However, their primary structures are not conserved among species, and their cDNA sequences are not easily cloned in non-model fish species by traditional homology cloning strategies. These sequence information will facilitate functional studies of cytokines in *Trachinotus ovatus*.

### SSR Discovery: Distribution and Frequencies

**Figure 7:**
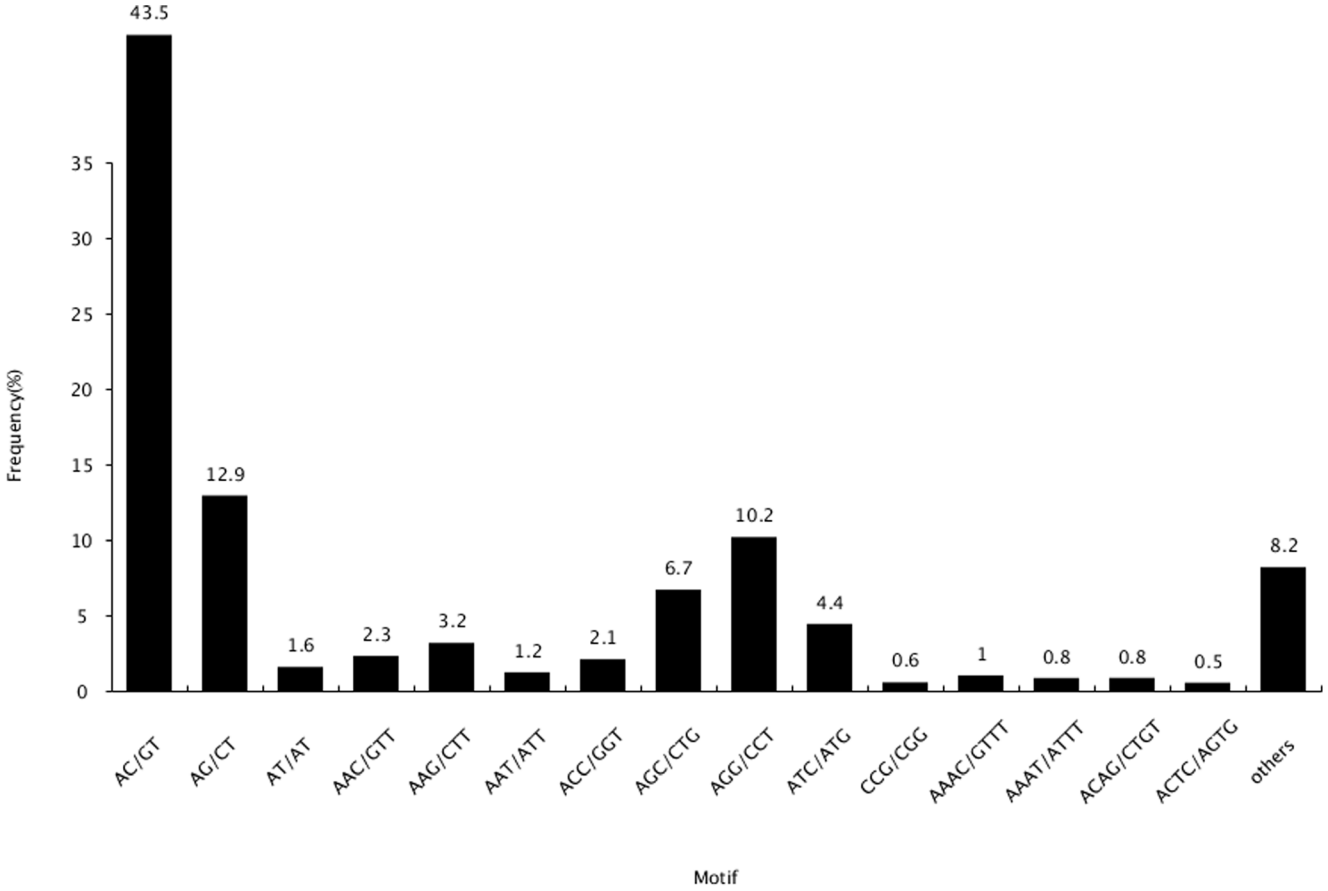
Frequency of classified repeat types of SSRs.

Simple sequence repeats, also known as microsatellites, are tandem repeats of 1–6 nucleotides found in all prokaryotic and eukaryotic genomes [61]. They have been widely used in genetic linkage map construction, population genetic studies, molecular marker-assisted breeding and so on [62]. As there is no genome sequence information, transcriptomes are a useful alternative for SSR markers discovery of in *Trachinotus ovatus*. In our study, all of assembled sequences were applied to exploit potential SSRs using MISA software [25]. In total, 38,794 SSRs were recognized in 30,295 sequences (19.41%), and 6,616 sequences (4.24%) contained more than one SSR. There were 2,118 SSRs present in compound formation. Di-nucleotide repeats were the most abundant SSR motif (58.07%), followed by tri- (31.19%), tetra- (7.60%), penta- (1.88%) and hexa- (1.27%) nucleotide repeats (Table 2). A summary on the frequency of SSRs with different numbers of tandem repeats were presented in Figure 7. SSRs with six tandem repeats occurred at the highest frequency (26.21%), followed by those with five tandem repeats (19.27%), seven tandem repeats (16.30%), and nine tandem repeats (11.90%). Among the di-nucleotide repeats, the AT/GT motif accounted for the majority of SSRs (43.5%), followed by the AG/CT di-nucleotide repeat motif (12.9%), and the AT/TA di-nucleotide repeat motif (1.6%). The most frequent motifs in the tri-nucleotide repeats were ACG/CTG (10.2%).

**Table 2:** Distribution of identified SSRs using the MISA software.

Motif	Repeat numbers												Total	%
	4	5	6	7	8	9	10	11	12	13	14	≥15		
Di-	0	0	6560	4079	3480	4610	3121	655	23	0	0	1	22529	58.07
Tri-	0	6299	3434	2235	116	0	5	1	1	1	2	4	12098	31.19
Tetra-	1701	1095	144	3	3	0	0	0	1	0	0	1	2948	7.60
Penta-	656	56	7	2	2	3	1	0	0	0	0	1	728	1.88
Hexa-	391	27	22	5	4	5	5	3	8	0	5	16	491	1.27
Total	2748	7477	10167	6324	3605	4618	3132	659	33	1	7	23	38794	100.00
%	7.08	19.27	26.21	16.30	9.29	11.90	8.07	1.70	0.09	0	0.02	0.06	100.00	-

### Polymorphism Test of SSR Markers

To detect the genetic polymorphism of these microsatellite markers in the transcriptome, 331 pairs of SSR primers were designed for validation. In our study, 290 primer pairs were successfully amplified using *Trachinotus ovatus* genomic DNA (File S8). A large number of these microsatellite-containing transcripts could be annotated, most of which were associated with the metabolic pathways and immune system. Using the 290 primer pairs, 30 *Trachinotus ovatus* fish were analyzed, and 16 highly polymorphic microsatellite loci were found (Table 3). The rate of polymorphic microsatellites isolated in this study was low, perhaps because the tested individuals came from the same fish farm. More polymorphic microsatellites may be developed if more geographically distant populations were examined. Of the 16 polymorphic markers, the number of alleles per locus ranged from 2 to 14, with an average of 5.8. The observed and expected heterozygosities ranged from 0.152 to 0.663 and from 0.337 to 0.848, with an average of 0.318 and 0.682, respectively. The PIC values of 13 microsatellite loci were higher than 0.5, while that of the other 3 loci were between 0.29 and 0.46. Amongst the 16 SSR markers developed, 10 were located in unigenes annotated with known functions. The mammalian counterparts of these unigenes are involved in the regulation of various important cellular processes. For example, CCAAT/enhancer-binding protein beta 2 is an important transcription factor that controls the expression of genes involved in immune responses [63]. Retinoic acid receptor responder protein 3 is thought to act as a tumor suppressor or growth regulator [64]. Fam40b is essential for the differentiation of murine embryonic stem cells [65]. The potential links between these expressed sequence tag-simple sequence repeats (EST-SSRs) and some interesting phenotypes should be explored in further study.

**Table 3:** Characteristics of 16 polymorphic microsatellite loci.

Unigene ID	SSRs	No. of alleles	Ho	He	PIC	BLASTX, sequence description
0102325	(TG)9	8	0.165	0.835	0.780	similar to KIAA1486 protein
0102780	(CA)9	3	0.663	0.337	0.292	No
0041000	(CTG)20	7	0.352	0.648	0.576	CCAAT/enhancer-binding protein beta 2
0096447	(GT)9	5	0.292	0.708	0.650	transforming protein RhoA-like
0141384	(AC)9	14	0.152	0.848	0.807	No
0095395	(TG)10	4	0.261	0.740	0.674	No
0120853	(CA)9	5	0.349	0.651	0.581	No
0118815	(CAGCTC)12	8	0.210	0.790	0.744	similar to expressed protein
0128444	(TG)10	4	0.281	0.719	0.655	No
0019285	(CA)9	5	0.257	0.743	0.682	E3 ubiquitin-protein ligase RNF220-like
0090156	(CAGCTC)12	7	0.226	0.774	0.728	serine/threonine protein kinase
0110112	(TG)9	2	0.492	0.508	0.375	No
0141617	(CA)10	4	0.352	0.648	0.570	retinoic acid receptor responder protein 3-like
0106655	(TG)9	4	0.457	0.543	0.460	cell adhesion molecule 4-like
0091717	(GT)9	4	0.335	0.666	0.589	protein FAM40B-like
0108204	(AC)10	10	0.246	0.754	0.716	golgin subfamily B member 1-like

Ho, observed heterozygosity; HE, expected heterozygosity; PHWE, Hardy-Weinberg probability; PIC, polymorphic information content.

However, although the remaining 6 polymorphic microsatellite loci were came from transcribed regions, their sequences showed no similarity to any known protein when BLAST searches were performed on nucleotide and protein database. A possible explanation is that these microsatellite-containing sequences belong to microsatellite-containing transcripts. Another possible explanation may be that some sequences may be derived from poorly conserved untranslated regions. EST-SSRs have been recognized to be conserved and have cross-species transferability [66]. Therefore, these transcriptome-derived microsatellite markers are of high value, and can be employed for *Trachinotus ovatus* population genetic studies and linkage map construction in the future.

## Conclusion

For the first time, the whole transcriptome was studied in a fish species belonging to the genus *Trachinotus*. A large number of genes related to reproduction, growth and metabolism, and immune response were identified in the transcriptome dataset, providing abundant genomic data for future studies on the molecular mechanisms behind important physiological processes in *Trachinotus ovatus*. At the same time, many microsatellites were discovered and 16 polymorphic microsatellite loci were developed in this study, which are useful markers for genetic studies and marker-assisted selection in *Trachinotus ovatus*.

## Supporting Information

Figure S1 **Relative mRNA amounts of DMRT1 in adult gonads of** ***Trachinotus ovatus*** **.**
(DOCX)

Table S1 **The functional classification of the COG classes for the transcriptome of** ***Trachinotus ovatus.***
(XLSX)

Table S2 **KEEG pathway analysis for the transcriptome of** ***Trachinotus ovatus.***
(XLSX)

Table S3 **GO terms associated with reproduction.**
(XLSX)

Table S4 **IGF/IGFR signaling pathways.**
(XLSX)

Table S5 **GO terms associated with response to stimulus.**
(XLSX)

Table S6 **GO terms associated with immune system process.**
(XLSX)

Table S7 **KEEG pathway analysis for immune response.**
(XLSX)

Table S8 **Information of 290 SSRs primer pairs derived from unigenes.**
(XLSX)

Text S1 **Putative sequences related to reproduction in the transcriptome of** ***Trachinotus ovatus*** **.**
(TXT)

Text S2 **Putative sequences related to growth and metabolism in the transcriptome of** ***Trachinotus ovatus*** **.**
(TXT)

Text S3 **Putative sequences related to immunity in the transcriptome of** ***Trachinotus ovatus*** **.**
(TXT)
