# Supplementary material for: Transcriptome Analysis of the Trachinotus ovatus: Identification of Reproduction, Growth and Immune-Related Genes and Microsatellite Markers
Source: PLoS One. 2014 Oct 10;9(10):e109419. doi: 10.1371/journal.pone.0109419 (PMC4193775; doi:10.1371/journal.pone.0109419)
Supplement: Figure S1 — Relative mRNA amounts of DMRT1 in adult gonads of Trachinotus ovatus . (DOCX) [file pone.0109419.s001.docx]

**Relative mRNA amounts of DMRT1 in adult gonads of *Trachinotus ovatus*:**


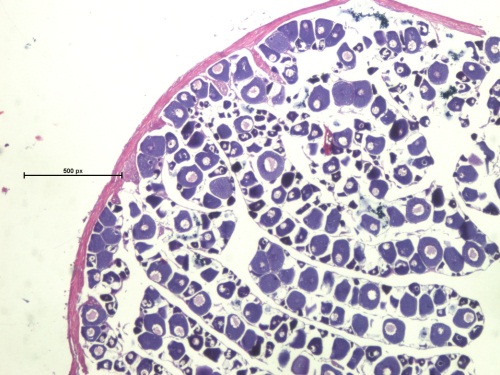

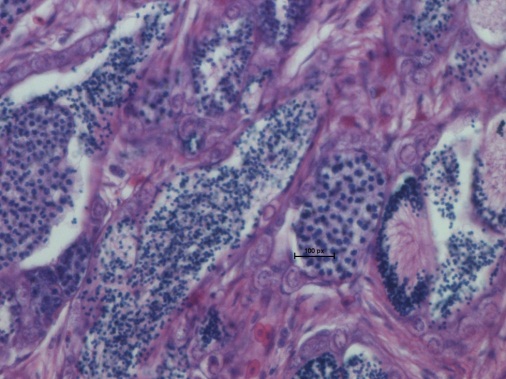

**Material and Method**

Total RNA from the gonad was prepared using Trizol reagent (Invitrogen). One microgram of isolated RNA was used to synthesize the first-strand cDNA using the ReverTra Ace-α First-strand cDNA Synthesis Kit (TOYOBO, Japan).

Real-time PCR was performed on a Roche LightCycler 480 real-time PCR system using the SYBR Green I Kit (TOYOBO, Japan) according to the manufacturer’s instructions. For each sample, 5 μL SYB Premix Ex TaqTM, 0.5 uL cDNA, 4.1 μL H_2_O, 0.2 μL of each forward and reverse primer were used for amplification. The reaction program was as following: an initial heating at 50℃ for 10 min, denaturation at 94℃ for 2 min, followed by 40 cycles at 94℃ for 15 s; 55 ℃ for 15 s and 72℃ for 30 s. Temperature for collecting fluorescence was set at 82℃. After amplification, a melting curve was gained using two additional cycles by reading the fluorescence value from 50℃ to 95℃. The standard curve of amplification for dmrt 1 or 18S rRNA genes was generated between cycle threshold (C_T_) value and the logarithm of vector dilutions using serial dilution (six-points) of quantified pTZ57R vector (MBI Fermentas, USA) containing the fragment of interest. Data analysis was performed via LightCycler software 480 (Roche, Swiss). The concentration of template in the sample was determined by relating the C_T_ value to the standard curve. Dmrt1 transcript levels were normalized against 18S transcript levels.

**Table Primers used for the realtime PCR**

| Primers | Sequence(5’to3’) |
| --- | --- |
| Dmrt1 F1 | CCCTGAGGTGATGGTGAAGAA |
| Dmrt1 R1 | ACAGCAAGCGTTGAAGATGAG |
| 18S F1 | CCTGAGAAACGGCTACCACATCC |
| 18S R1 | AGCAACTTTAGTATACGCTATTGGAG |
